# Pearle’s Hidden-Variable Model Revisited

**DOI:** 10.3390/e22010001

**Published:** 2019-12-18

**Authors:** Richard David Gill

**Affiliations:** Mathematical Institute, Leiden University, PO Box 9512, 2300 RA Leiden, The Netherlands; gill@math.leidenuniv.nl

**Keywords:** Bell’s theorem, detection loophole, computer simulation, Pearle’s model

## Abstract

Pearle (1970) gave an example of a local hidden variables model which exactly reproduced the singlet correlations of quantum theory, through the device of data-rejection: particles can fail to be detected in a way which depends on the hidden variables carried by the particles and on the measurement settings. If the experimenter computes correlations between measurement outcomes of particle pairs for which both particles are detected, he or she is actually looking at a subsample of particle pairs, determined by interaction involving both measurement settings and the hidden variables carried in the particles. We correct a mistake in Pearle’s formulas (a normalization error) and more importantly show that the model is simpler than first appears. We illustrate with visualizations of the model and with a small simulation experiment, with code in the statistical programming language R included in the paper. Open problems are discussed.

## 1. Introduction

Bell’s (1964) landmark paper [1] “On the Einstein Podolsky Rosen paradox” led a few years later to a version of his inequality more suitable for experimental purposes, and consequently the focus of a very great deal of both experimental and theoretical work. That is the inequality nowadays called the Bell-CHSH inequality, presented by Clauser, Horne, Shimony and Holt (1969) [2]. Almost immediately, however, Pearle (1970) [3] pointed out that the problem of detector efficiency meant that it was easy under local realism to reproduce the famous negative cosine curve of the correlations between spin measurements on particles in the singlet state. The measurements on each particle would not have two outcomes but three: spin up, spin down, and no detection. One would be tempted to restrict attention to only those “trials” in which both particles were detected, and compute the correlation between the observed spins for that subpopulation. Whether or not a particle was detected was, in Pearle’s model, determined by a hidden variable correlated with the actual “hidden” spins of the particles. Detection depended on the extra hidden variable and on the detector setting. Selection of particle pairs such that both particles got detected effectively selects a subpopulation of particle pairs, whose hidden spins actually depend on the detector settings.

This would result in experimental violation of the CHSH inequality, moreover with the maximal violation predicted by quantum mechanics, even though there is a perfect local realistic explanation of the correlations found.

Pearle’s model is the subject of this paper. It was the starting shot in a huge literature on the detection loophole, which continues to grow to this day. Pearle’s model did have some unphysical features, and he was well aware of them. In his model, the probability of a double detection would depend on the angle between the two detectors and hence the experimenter would immediately notice that his or her results did not make sense. The paper was for many years considered a purely theoretical exercise which established a purely theoretical lower limit to detector efficiency which would have to be exceeded before a so-called loophole-free experiment could be carried out. Soon, other detection loophole models were discovered which did not have his model’s defect. Later, models were found that also established the same lower limit, and it was also shown that the bound was optimal.

Since the literature is so huge, it cannot be adequately surveyed in this paper, and I refer the reader to the most recent comprehensive survey (Larsson (2014) [4]). That paper in fact covers all of the “known” loopholes, not just the detection loophole. A year later, in 2015, the first “loophole-free” experiments were performed and experimental violation of appropriate Bell-type inequalities observed. However, the detection loophole remains of great interest and new detection-loophole models are continually being invented. In fact, many, both old and new, can even be considered as variations on Pearle’s. The purpose of this paper is to clarify this situation, and also to make Pearle’s work more accessible. His paper is unfortunately marred by curious notational conventions, confusing misprints, and some real errors in key formulas (incorrect normalization constants). It seems that these errors have not been noticed before. In fact, as far as I know, nobody had actually tried to implement Pearle’s model in simulation programs before. (Philip Pearle himself, private communication, was also not aware of any implementation).

I only mention a very small number of other key papers. Regarding the early years, the landmark paper by Clauser and Horne (1974) [5] already includes another detection-loophole model without the just mentioned bad feature of Pearle’s. More recently, Gisin and Gisin (1999) [6] came up with another. An important survey and many new results were provided by Garg and Mermin (1987) [7]. A little known but very interesting survey was provided by Risco-Delgado (1993) [8]. A whole series of important contributions was made by Jan–Åke Larsson (see his 2014 survey paper [9]); of particular relevance to the detector efficiency issue are the papers by Larsson (1998) [9] and Larsson and Semitecolos (2001) [10].

Technical note: this paper has been composed in the *R markdown language* and typeset using the *R* package “knitr” and *RStudio*, a popular IDE for working with *R*. The source code contains therefore interleaved passages of text (including LATEX code, for instance, for mathematical formulas) and *R* code. Processing the original *Rmd* file with knitr generates a LATEX source file containing interleaved text, *R* code and *R* textual output. It also generates pdf figures of *R*’s graphical output. Most readers will not be interested in the R code. It can easily be skipped; hopefully the figures are well enough explained in the surrounding text.

## 2. Pearle’s Model Simplified

**Figure 1:**
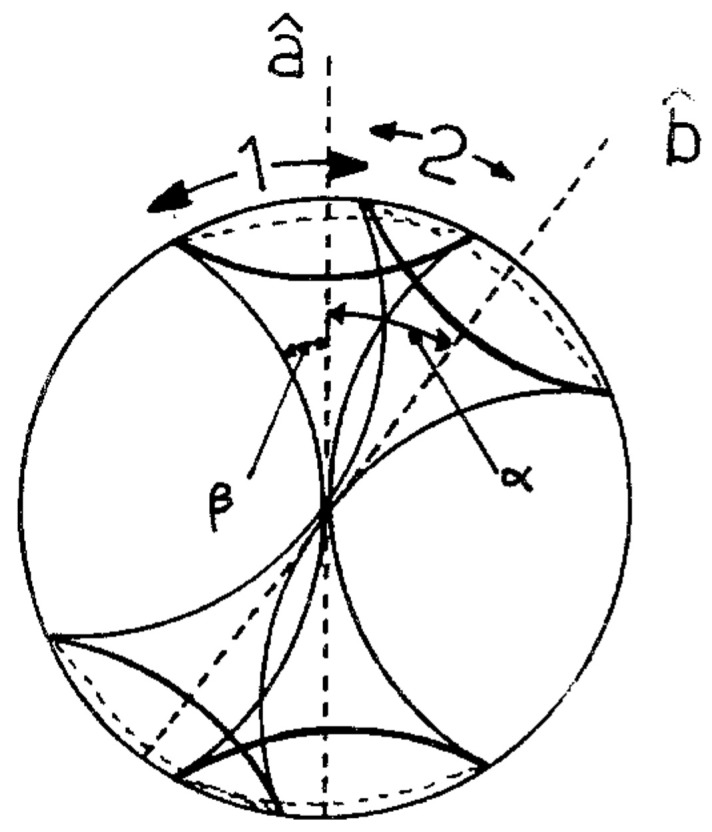
Pearle’s sphere as portrayed by Risco-Delgado.

Pearle’s model is best understood with the help of a picture. Figure 1 is taken (and used with the author’s permission) from Risco-Delgado (1993) [8].

Risco-Delgado explains as follows: “Pearle’s sphere represents the nine possible outcomes of an EPR experiment allowing undetected events. If the representative point lies in region 1, the particle A will be measured as possessing spin parallel to $\hat{a}$, and the particle B will not be detected. If it lies in region 2 the particle the particle A will be measured as possessing spin parallel to $\hat{a}$ and particle B antiparallel to $\hat{b}$, and so on. There exist nine possibilities defending on the position of the point and the relative angle α between $\hat{a}$ and $\hat{b}$.”

Pearle and Risco-Delgado are modeling source emitting pairs of particles. The particles carry hidden variables X and Y which we take to be random points in the unit ball in $R^{3}$. We assume that Y=−X and X≠0 with probability 1. The ball is drawn in the figure.

Write X=RU where ${∥U∥}^{2}=1$ and R>0. One might think of the unit length vector U as the *direction* of spin of the first particle, and −U as the direction of spin of the second, equal and opposite points on the unit sphere $S^{2}$, while the scalar *R* is some kind of amplitude of spin.

Assume that the direction U is uniformly distributed on $S^{2}$ and statistically independent of the amplitude R∈(0,1].

Notation: bold (as opposed to italic) indicates a vector; random vectors and random variables are denoted by uppercase symbols, while lowercase is used for non-random quantities.

Each particle gets measured in directions a and b, respectively (points on $S^{2}$, chosen freely by the experimenter); these were the directions $\hat{a}$ and $\hat{b}$ in the figure. The possible outcomes are +1 (“spin up”), −1 (“spin down”), and “no detection”, according to the following rule: if the angle between X and a is less than Rπ/2, then the outcome of measuring the first particle is +1; if the angle between X and −a is less than Rπ/2, then the outcome of measuring the first particle is −1; otherwise, the particle is not detected at all. The rule for the second particle is exactly the same story as for the first particle, with X and a replaced throughout by Y and b.

The smaller of the angles between X and ±a is $\cos^{-1}\left|U\cdot a\right|$ so the recipe becomes: the outcome of measuring the first particle is sign U·a if $\cos^{-1}\left|U\cdot a\right|\geq R\pi /2$ while there is “no detection” if $\cos^{-1}\left|U\cdot a\right|<R\pi /2$; the outcome of measuring the second particle is −sign U·b if $\cos^{-1}\left|U\cdot b\right|\geq R\pi /2$ while it is not detected if $\cos^{-1}\left|U\cdot b\right|<R\pi /2$.

Pearle (1970) gives a formula, Equation (22) in his paper, for a particular choice of the probability density of *R*; however, take note of his idiosyncratic normalization (Equation (1))! There is an error in his derivation, as can be verified by integrating the density over the whole range: combining Equations (1) and (22), we get a density which does not integrate to 1. Working through Pearle’s paper in detail, it turns out that the only error in Equation (22) is the normalization constant, and this probably derives from an incorrect normalization in Equation (14) where Pearle switches from *R* to S=cos(Rπ/2), but it is difficult to be certain about this, since his notion of probability density is ambiguous and unconventional.

Here, I present an alternative and much simpler description of the distribution of *R* and also of the whole model, via the distribution of S=cos(Rπ/2)∈[0,1). It turns out that the distribution of *S* can be expressed by the formula $S=(2/\sqrt{V})-1$ where V∼Unif(1,4); and moreover it is *S* that we primarily need to know in order to simulate the model.

In terms of $S=(2/\sqrt{V})-1$, the recipe for simulating the measurement of one pair of particles is as follows: generate U uniformly at random on the sphere $S^{2}$ and independently thereof, generate *V* uniformly at random in the real interval [1,4]. Compute $S=\left(2/\sqrt{V}\right)-1\in \left(0,1\right)$, A=U·a, and B=−U·b. Particle 1 is detected if and only if |A|≥S, and, if it is detected, the outcome of measurement is sign(A). Particle 2 is detected if and only if |B|≥S, and, if it is detected, the outcome of measurement is sign(B).

Pearle’s main result is that this model reproduces the singlet correlations:

E(sign(A)sign(B)||A|≥S and |B|≥S) = −a·b.

I do not reproduce Pearle’s (magnificent but of necessity very involved) proof. Instead, I just derive the density of *R* according to my specification, so that the reader can compare with Pearle’s formula. I then “prove” Pearle’s result by a simulation experiment. In fact, I would dearly like to see a short-cut derivation of Pearle’s result. Through some quite brilliant calculations, he characterizes all possible probability distributions of *R* (equivalently, of *S*) which reproduce the singlet correlations as (up to normalization) the positive functions within the range of a certain differential operator, and then shows that the operator when applied to the constant function—the most simple choice one could make—is indeed positive. Further details are given in Appendix A at the end of this paper.

According to my definitions, $R=\cos^{-1}\left(S\right)/\left(\pi /2\right)$ and it follows that for r∈(0,1),

$$\begin{array}{cc} \mathrm{Pr}\left(R\leq r\right)= & \mathrm{Pr}(S\geq \cos\left(r\frac{\pi }{2}\right))=\mathrm{Pr}\left(2/\sqrt{V}-1\geq \cos\left(r\frac{\pi }{2}\right)\right)\\ & \;=\mathrm{Pr}\left(\sqrt{V}\leq \frac{2}{1+\cos(r\frac{\pi }{2})}\right)\\ & \;=\mathrm{Pr}\left(V\leq \frac{4}{{\left(1+\cos\left(r\frac{\pi }{2}\right)\right)}^{2}}\right)\\ & \;=\frac{1}{3}\left(\frac{4}{{\left(1+\cos\left(r\frac{\pi }{2}\right)\right)}^{2}}-1\right) \end{array}$$

and hence the probability density of *R* is

$$\begin{array}{cc} f_{R}\left(r\right) & =\frac{4}{3}\cdot 2\cdot \frac{\pi }{2}\cdot \frac{\sin(r\frac{\pi }{2})}{{\left(1+\cos\left(r\frac{\pi }{2}\right)\right)}^{3}}\\ & =\frac{4\pi }{3}\cdot \frac{\sin(r\frac{\pi }{2})}{{\left(1+\cos\left(r\frac{\pi }{2}\right)\right)}^{3}} \end{array}$$

on the interval (0,1). Compare this to Pearle’s Equations (1) and (22) combined:

$$4\pi \rho \left(r\right)r^{2}\;=\;\frac{16}{3}\cdot \frac{\sin(r\frac{\pi }{2})}{{\left(1+\cos\left(r\frac{\pi }{2}\right)\right)}^{3}}.$$

**Figure 2:**
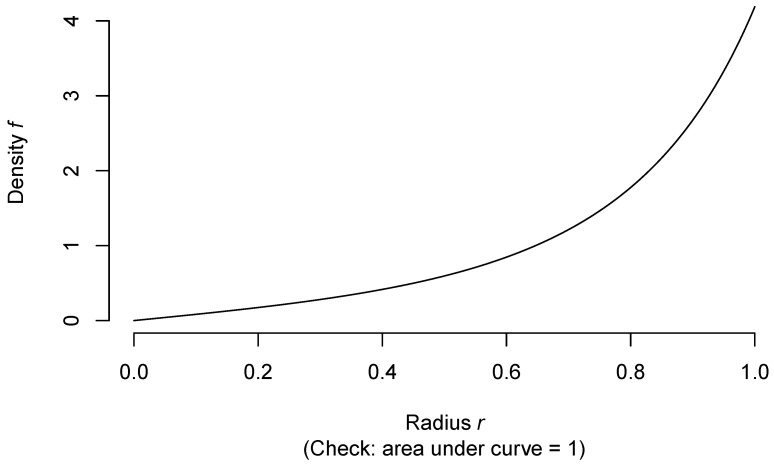
Probability density of *R*.

The code below generates a graph, Figure 2, of the probability density of *R*, as well as making a rough numerical check that it integrates to 1. Since the probability density is monotone increasing, we get guaranteed lower and upper bounds to the integral by summing the value of the density on a regular grid of points between 0 and 1, omitting the right hand and left hand endpoints, respectively, and dividing by the number of intervals generated by the grid.


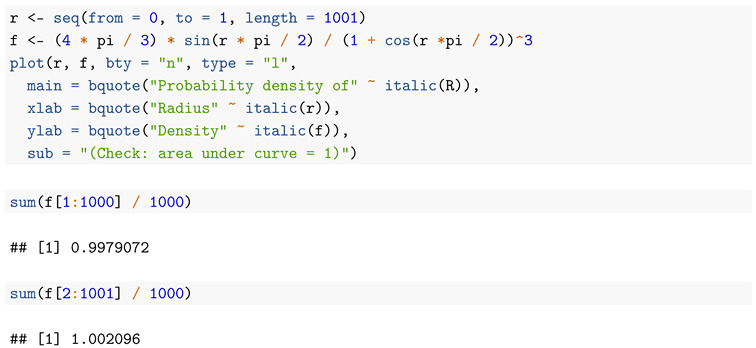


**Figure 3:**
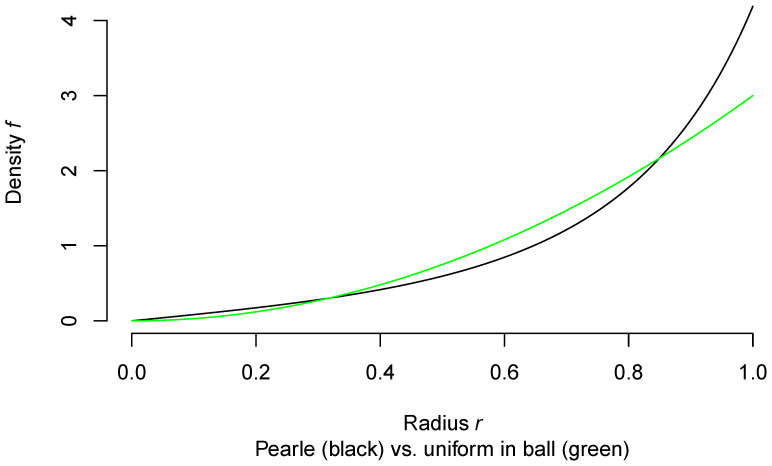
Comparison of two models for density of *R*.

If the points *X* had been chosen uniformly distributed within the unit ball, the probability density of their distance *R* to the origin would have had probability density $3r^{2}$, 0≤r≤1. In Figure 3, I compare the two densities (the one corresponding to a uniform distribution over the ball in green).


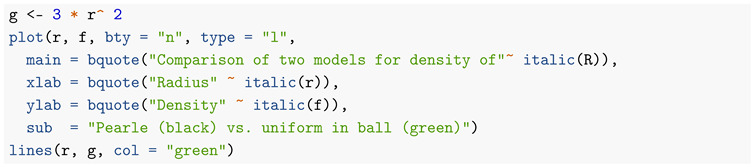


We see that Pearle’s points have a tendency to be closer to the surface of the ball than if they had been uniformly distributed throughout it.

According to Pearle’s model, Particle 1 is represented by a point in the ball. It is *observed* when its spin is measured in a certain direction, if and only if its point lies in either of two “mushroom shaped” regions around the measurement direction and its opposite. It ±1 depending on the region. Particle 2 is represented by the exactly opposite point in the ball but for the rest, its observation and measurement follow exactly the same rule, with respect to its own two mushrooms. Thus, if both particles are measured in the same direction, either neither is observed or both are observed, and if observed, the two outcomes are certain to be opposite. Measured in opposite directions, either neither is observed or both are observed, and if observed the two outcomes are certain to be the same.

**Figure 4:**
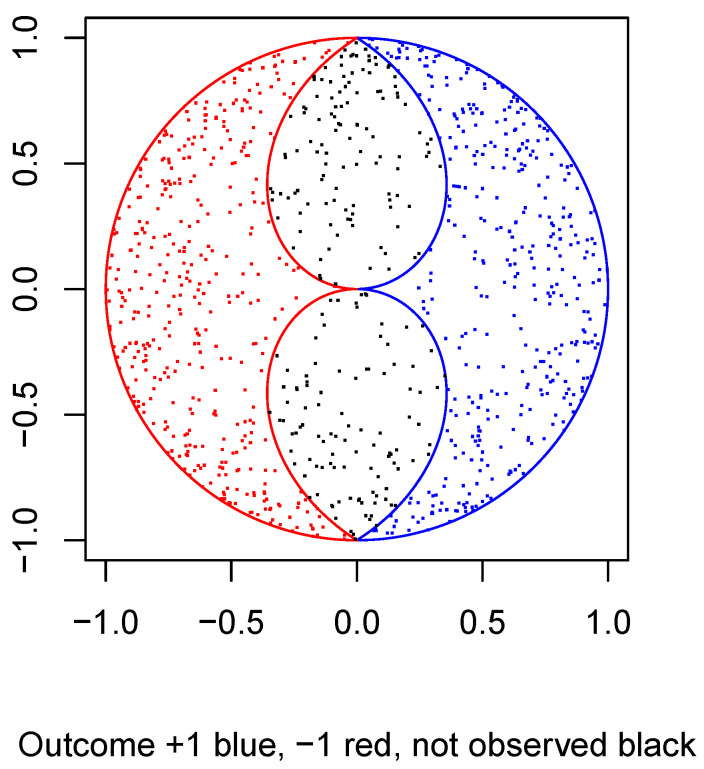
Pearle’s sphere.

In the following plot, Figure 4, I draw the intersections of the two mushrooms for Particle 1 with a plane through the origin containing its measurement direction, which is taken to be the direction of the positive *x*-axis. I superimpose on this plot a sample of 1000 particles distributed in a circularly symmetric way about the centre of the unit *disk*, with distance to the origin distributed as in Pearle’s model. The result is a 2D *caricature* of Pearle’s 3D model: some statistical features are the same, some are different.

The picture is neither a 2D section nor a 2D projection of the 3D model. However, it should help the reader to visualize the model. The points are colored blue, red, or black according to whether the corresponding particle measurement result is an outcome spin up, spin down, or the particle is not detected. Two particles are simultaneously measured in this way: same point in the ball, different directions of measurement, altogether four mushrooms.

The actual detection regions (the red and the blue mushroom) are formed by rotating the 2D boundaries about the *x*-axis. The actual distribution of the 3D hidden variable of the particles being measured has the same radial component as in the plot, but its direction is now uniform over the sphere, instead of the circle. Thus, the 3D density of points is less than what the picture suggests as we move further from the origin; however, it still increases as we move outward relative to a uniform density.


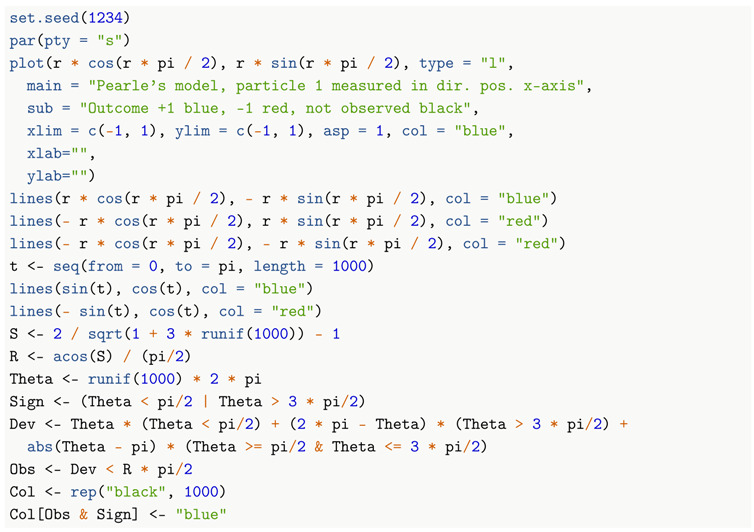



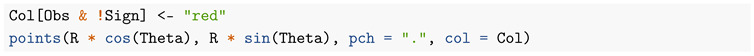


Pearle found his model by fixing the mushroom shape first, and then looking for a probability distribution of the radial distance *R* such that the pair (mushroom shape, distribution of *R*) reproduce the singlet correlations. If we transform the unit ball onto itself in a continuous way by applying a monotone transformation of the unit interval onto itself to the distances of points from the origin, we can transform the distribution of *R* into any distribution on [0,1] with cumulative distribution function which is continuous and strictly increasing throughout the interval. The mushroom shape will be transformed correspondingly. The author of this paper has not discovered a transformation which simultaneously makes both the the mushroom shape and the distribution of *R* more simple than what they are at present. In fact, Pearle’s choice does amount to fixing one of these two coupled parameters so that it has a direct physical interpretation: a particle pair with a particular value *r* of *R* is such that each particle is not detected at all if the direction of its spin, thought of now as an undirected line through the origin, deviates by more than rπ/2 from the direction in which the spin is measured, also thought of an undirected line through the origin.

Altogether, Pearle’s derivation of his model was a tour-de-force in imagination, analysis, and geometry. Whether or not there is a short-cut to getting his results and whether or not they can be improved are interesting challenges. As we see in the next section, the model has one major defect, namely the rate at which a pair of particles are both detected depends quite strongly on the pair of settings with which they are measured. This phenomenon would be experimentally observable; conversely, the usual quantum mechanical modeling of this experiment, and assuming that particles are detected independently of the direction in which their spin is measured, predicts that the rate of pair detection is independent of measurement settings. Thus, we are left with the open problem: Is there a distribution of *R* reproducing the singlet correlations which does not have this defect? Pearle does not answer this question explicitly but his text suggests that he believes the answer is negative. A numerical analysis (see Appendix A) confirms.

## 3. A Simulation Experiment

We now present a simulation of the model in the statistical programming language “R”. First, we (re)set the random seed, for reproducibility. To see results based on a fresh sample, replace the (integer) seed by your own, or delete this line and let your computer dream up one for you (it uses system time + process ID to do this job).


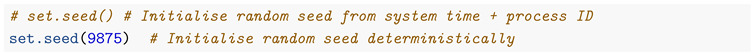


We generate uniform random points on sphere generated using the “trig method” (Method 3) of Dave Seaman: see http://rpubs.com/gill1109/13340 for an R illustration. This very effective but little known method uses the *coincidence* that in 3D, a uniform point on the sphere has a *z* coordinate which is uniformly distributed between −1 and +1. Thus, we proceed as follows.

(a) Choose *Z* uniformly distributed in [−1,1].
(b) Choose Θ uniformly distributed on [0,2π).
(c) Let $R=\sqrt{(}1-Z^{2})$.
(d) Let X=Rcos(Θ).
(e) Let Y=Rsin(Θ).

In the following simulation, the measurement directions are all in the equatorial plane, so only *Z* and *X* have been generated and are treated as *X* and *Y*.

First, we set up the measurement angles for setting “a”: directions in the equatorial plane.


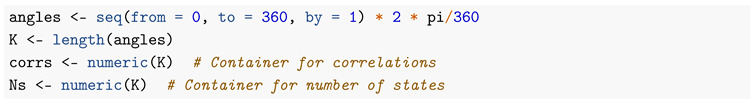


For setting “b”, we use a fixed direction.


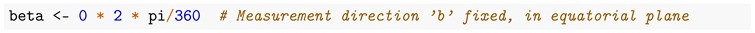


Then, we set the sample size (number of pairs of particles).


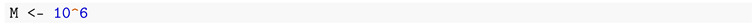


I use the same, single sample of $M=10^{6}$ realizations of hidden states for all measurement directions.


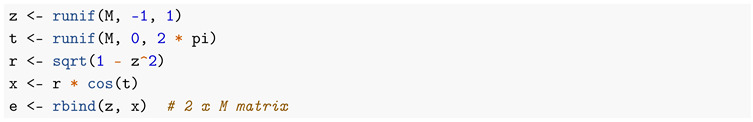


The *M* columns of *e* represent the *x* and *y* coordinates of *M* uniform random points on the sphere $S^{2}$.


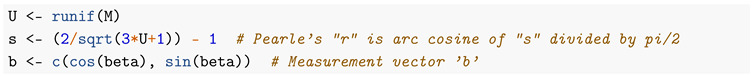


Loop through measurement vectors “a” (except last = 360 degrees = first):


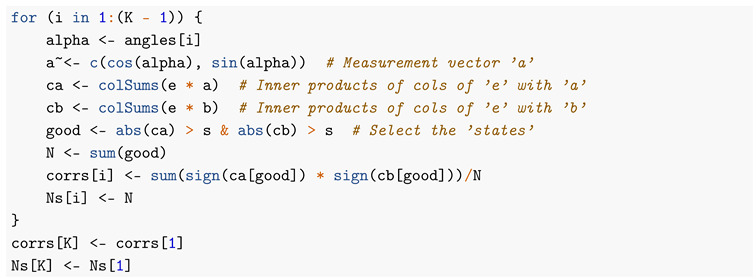


**Figure 5:**
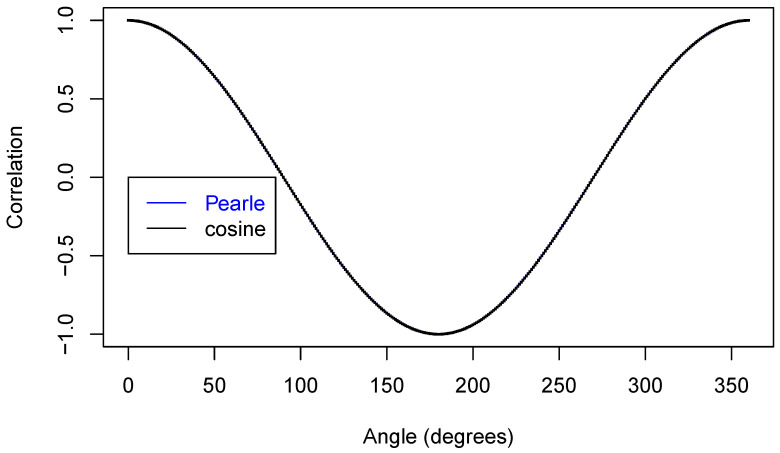
Two correlation functions.

Now, we are ready to make some plots of the results. First, Figure 5 shows that we have nicely reproduced the theoretical cosine.


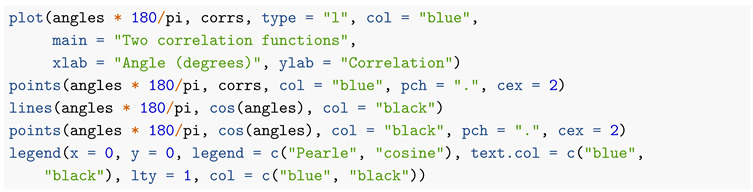


**Figure 6:**
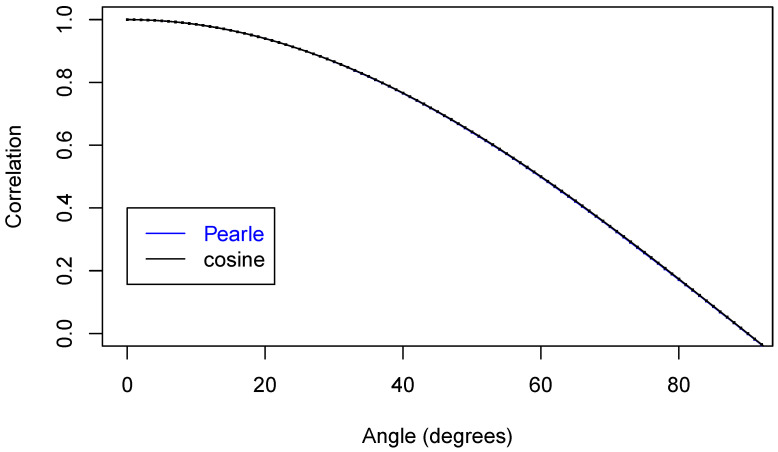
Two correlation functions.

In Figure 6, we zoom in on just part of the curve.


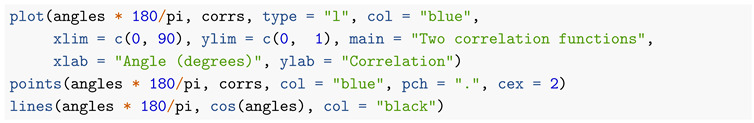



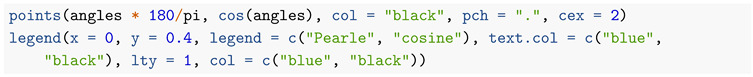


**Figure 7:**
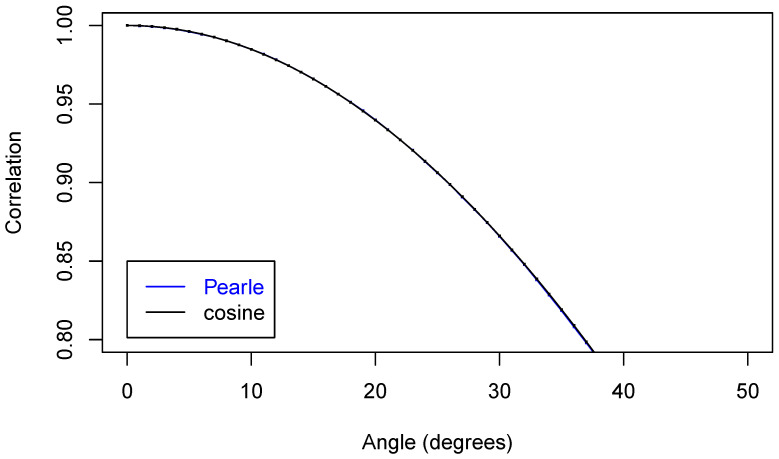
Two correlation functions.

In Figure 7 is an even closer look at part of the curves.


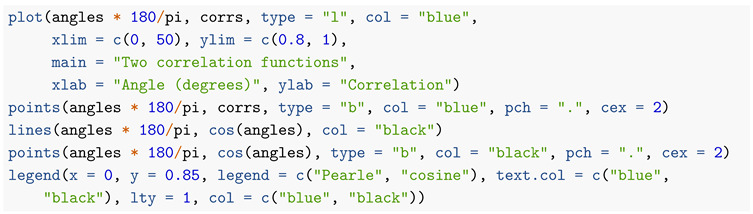


**Figure 8:**
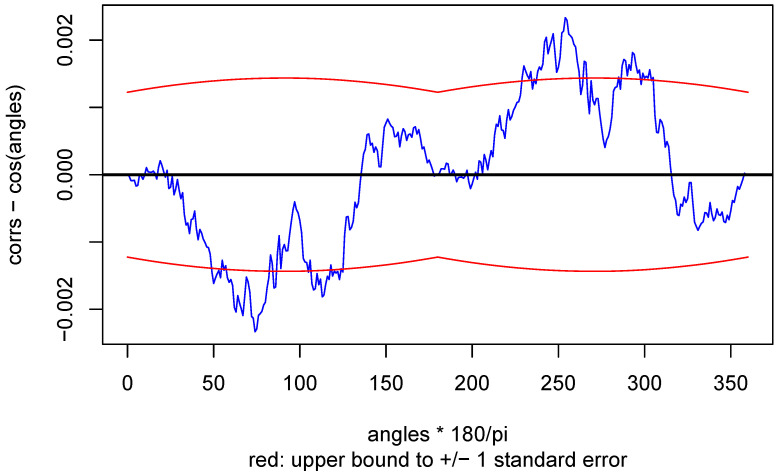
Difference.

Next, Figure 8 is a plot of the differences between theory and simulation, with an indication of accuracy.


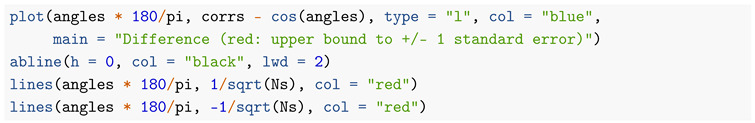



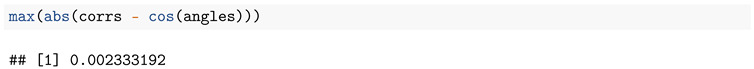


**Figure 9:**
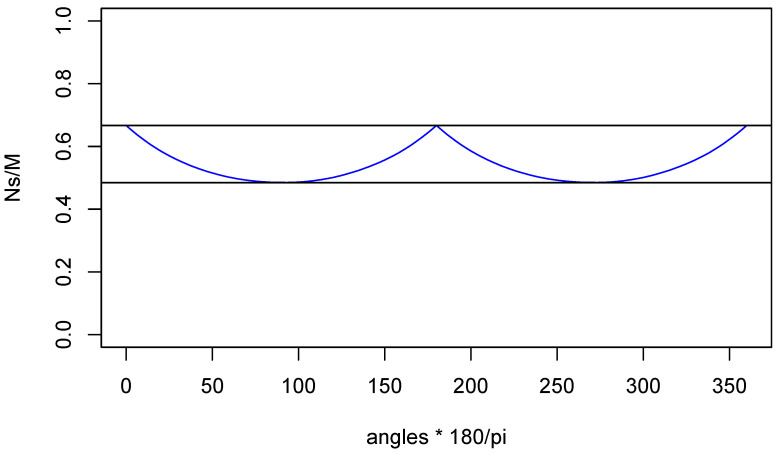
Rate of detected particle pairs.

Finally, Figure 9 is a plot of the proportion of observed particle pairs to emitted pairs, as function of the angle between the measurement directions.


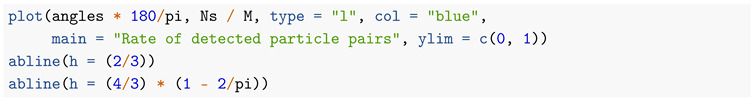


The two horizontal lines are the maximum and minimum detection rates computed by Pearle: 2/3 and 4/3(1−2/π)=0.4845… of *M*, respectively. Now, if an experimenter is not using pulsed emission of particle pairs but they are being emitted in a continuous fashion according to a Poisson process, then the experimenter will have no way of knowing that, when neither particle is detected, there was still an emission of a particle pair. Thus, the loss of 1/3 of all emitted particle pairs will go unnoticed. However, the experimenter will be able to see that the rate of double detections depends strongly on the difference between the two measurement directions—the maximum rate is more than 4/3 times the smallest. Put another way: The rate at which particles are detected at one measurement station with no accompanying detection at the other depends on the difference between the two measurement directions. Thus, Pearle’s model has some very unsatisfactory features: assuming a constant emission rate, the experimenter can see that particles are suspiciously being rejected in a way which depends on both the settings. It was only in 2008 that Gisin and Gisin [6] came up with a new local hidden variable model for the singlet correlations based on data rejection which possesses *all* the symmetries one would require. Moreover, it is amazingly simple. However, it seems further from physical interpretation than Pearle’s model.

However, Pearle did more than exhibit one concrete local hidden variable model, which reproduces the singlet correlations: he also characterizes the class of all distributions of *R* which does the job. This allows us in principle to find out if there is a distribution within the class which leads to a model with all required symmetries. I believe the answer is negative (and I believe that Pearle believed this too) but I do not have a mathematical proof. Numerical evidence (see Appendix A) is very strong and inspection of the numerical result might help in constructing a proof. 

## Figures and Tables

**Figure A1:**
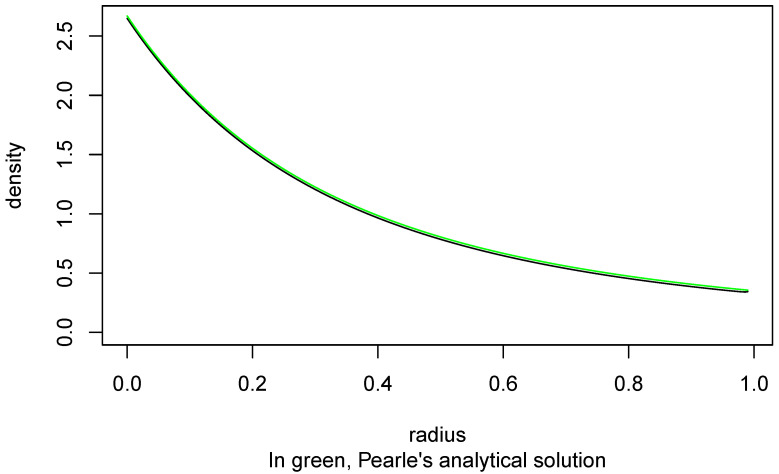
Solving for density numerically, with μ = constant.

**Figure A2:**
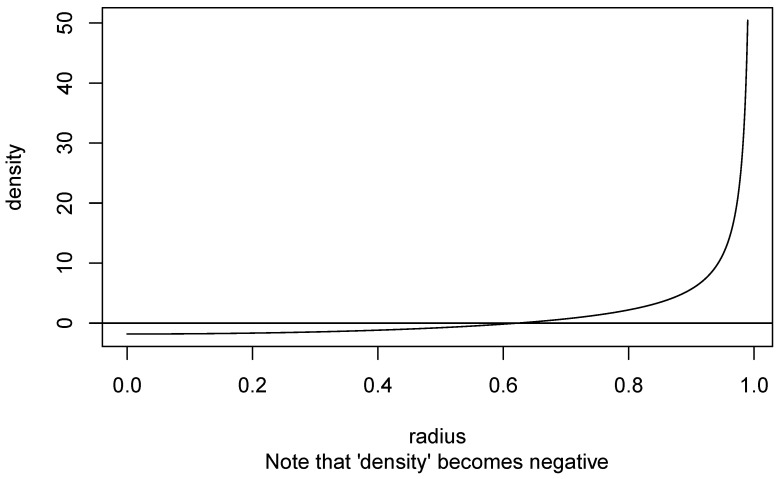
Solving for density numerically, with *g* = constant.

## Appendix A

Here, I reproduce Pearle’s description of the class of *all* distributions of *R* such that the singlet correlation is recovered from the measurement outcomes of detected particle pairs.

Let μ be a real function on [0,1] satisfying the symmetry requirement $\mu \left(x\right)=\mu ({\left(1-x^{2}\right)}^{1/2})$ for all *x*. One could for instance pick μ arbitrarily on $[0,1/\sqrt{2}]$ and use the symmetry requirement to determine μ on $\left(1/\sqrt{2},1\right]$. It would probably be wise to impose continuity of the derivative of μ at $1/\sqrt{2}$. Next, compute the function *h* through

$$h\left(x\right)\;=\;\frac{d^{2}}{dx^{2}}\left(\frac{x^{2}}{1-x^{2}}\left[\int_{0}^{1}{\left(1-z^{2}\right)}^{\frac{1}{2}}\mu \left(z\right)dz-\int_{0}^{x}{\left(1-\frac{z^{2}}{x^{2}}\right)}^{\frac{1}{2}}\mu \left(z\right)dz\right]\right).$$

If *h* is nonnegative and integrable, normalize it to a probability density: this should be the probability density of S=cos(Rπ/2). The choice μ= constant delivers the particular distribution of *S*, which Pearle further investigates and which we have studied here.

I am not aware of a simple interpretation of the function μ so its role is hard to understand. It is the result of applying a certain differential operator, Pearle’s Equation (17)

$$\mu \left(x\right)\;=\;-\frac{1}{{\left(1-x^{2}\right)}^{1/2}}\frac{d}{dx}\left[\frac{{\left(1-x^{2}\right)}^{2}}{4x}\frac{d}{dx}(g\left(x\right)x^{2})\right]$$

to the function *g* defined as the probability of detection of a particle pair, expressed as function of s=cos(rπ/2).

In principle, we can therefore see what happens if we specify g= constant, and giving $\mu \left(x\right)=Cx{\left(1-x^{2}\right)}^{1/2}$; this results in a candidate for the function *h* and we only have to find out whether or not *h* can be normalized to a probability density (integrable, nonnegative). I was not able to perform this operation analytically. However, numerical integration and differentiation delivers us a candidate *h* which takes both negative and positive values. This provides evidence that his model does *not* include a distribution for *R* (or equivalently *S*) such that the pair detection probability is independent of the settings. The numerical analysis did moreover confirm the theoretical analysis for the case μ= constant.

The investigation is hampered by the misprints in Pearle’s paper: for instance, the power in Equation (21) should be −3 not +3, and throughout, normalization constants are not to be trusted. The following very naive code “computes” the density of *S* first in the case of constant μ, see Figure A1, and then in the case of constant *g*, Figure A2.
